# Supplementary material for: Protein engineering and iterative multimodule optimization for vitamin B6 production in Escherichia coli
Source: Nat Commun. 2023 Aug 31;14:5304. doi: 10.1038/s41467-023-40928-0 (PMC10471632; doi:10.1038/s41467-023-40928-0)
Supplement: Supplementary file 1 — Supplementary information [file 41467_2023_40928_MOESM1_ESM.pdf]

Protein engineering and iterative multimodule optimization for vitamin B_6 production in *Escherichia coli*

Liu *et al*.

Supplementary Method 1. Proteomic cultures preparation, digestion, and proteomic analysis

Strains used in this study are listed in Supplementary Data 4. Cells were cultivated at 37 °C in the fermentation medium FM1.4 to log phase at 250 mL shake flask and 5L bioreactor. Samples were harvested based on the OD_{600} and centrifugated at 1500 g for 5 min. Cell pellet was washed with pre-cold phosphate buffer saline (PBS) and lysed in lysis buffer (8 M urea dissolved in 50 mM Tris-HCl with protease inhibitor cocktail (Roche) by Zirconia/Silica beads beating at -50 °C. Next, the cell lysate centrifuged at 15,000 g for 20 min. The supernatant was diluted and the concentration of protein was determined by BCA protein assay kit.

The method of liquid chromatography and mass spectrometry were performed according to the previous study with modification [1,2]. Peptide was carried out on a nanoElute liquid chromatography system (Bruker Daltonics). Digested peptides were separated within 60 min at a flow rate of 350 nL/min on a homemade column 25 cm×75 µm, 1.9 µm C18-AQ particles (Dr. Maisch). Mobile phases A and B were water and ACN with 0.1% formic acid, respectively. The %B was linearly increased from 5 to 32% within 50 min, then increased to 95% within 6.9 min, and finally maintained at 95% for the last 3.1 min[1]. All samples were analyzed on a hybrid TIMS quadrupole time-of-flight mass spectrometer (Bruker timsTOF Pro2) via a CaptiveSpray nanoelectrospray ion source. The mass spectrometer was operated in data-dependent mode for sample analysis. The accumulation and ramp time were set at 100 ms each and the mass spectra were recorded in the range from m/z 100 to 1700 in positive electrospray mode. The ion mobility was scanned from 0.6 to 1.5 V s/cm^2. We acquired dda-PASEF with four PASEF scans per top-N acquisition cycle, an intensity threshold of 1000 arbitrary units (a.u.) as indicated by the Bruker acquisition software and a target value of 6000 a.u. for precursor selection. Precursors that reached this target value were excluded for 0.4 min. Singly charged precursors were filtered out based on their m/z-ion mobility position, and precursors with a mass below 700 Da were isolated with a quadrupole selection

window of 2 Th and otherwise with 3 Th.

Data was analyzed by MaxQuant Software (Version2.1.4.0)[3]. We used the reviewed *Escherichia coli* (strain K12) proteome (UniProt, Apr 2020,4480 entries without isoforms) and default settings for the analyses. This comprised a false discovery rate (FDR) of 1%, two missed cleavages, the cleavage pattern for trypsin, cysteine carbamidomethylation as fixed modification, and methionine oxidation and protein N-terminal acetylation as variable modifications. For the analysis of proteomic data, iBAQ and LFQ were used. The mass tolerance was set to 10 ppm for the main search, and known contaminants were excluded from the dataset [4].

Supplementary Method 2. Molecular dynamics (MD) simulations

All atom MD simulations were performed using the AMBER18 MD package (http://ambermd.org/, 2018). The bonded and non-bonded description of the interactions between the various atoms were generated using the AMBER18 force fields, which include the ff14SB force field parameters. The ANTECHAMBER module and GAFF2 with AM1-BCC charges were used to obtain force field parameters for ligands [5]. Initially, we performed a series of energy minimization steps to eliminate any faulty contacts in the initially built structures. During the minimization, protein (@CA, O, N, C) were restrained with harmonic force constants of 20 kcal/mol. The minimization process involved 5000 steps of steepest descent followed by 5000 steps using the conjugate gradient method. After the energy minimization, the system was slowly heated up to 310 K over 100 ps of MD using a 1 fs integration time step, while restraining the solute with a harmonic force constant of 20 kcal/mol. After this, we performed 50 ps of NPT equilibration of the structures with no harmonic restraints. Then, 20 ns constrained MD simulations were executed, so that the ligand was in a reasonable position to react. Finally, 100 ns NPT production simulations were performed at 310 K and 1 atm pressure with a 2 fs integration time step. We implemented periodic boundary condition across the system using a TIP3P water box [6]. We used the Particle Mesh Ewald (PME) techniques integrated with the AMBER package to account for the long range component of the electrostatic interactions [7]. During the dynamics, all the bonds involving hydrogen were restrained using the SHAKE algorithm [8]. A Langevin thermostat with collision frequency of 1/ps was used to maintain the constant temperature while the pressure was controlled using an anisotropic Monte-Carlo barostat [9]. The accelerated GPU version of PMEMD was implemented on NVIDIA GeForce 10 Series cards [10]. We employed the CPPTRAJ [11] functionality of AMBERTOOLs to perform various analyses on the equilibrium MD simulation trajectories.

Supplementary Discussion 1. Binding energies for the tested species

The binding energies for the substrate and coenzyme separately in the pre-catalytic state were calculated using the MMGBSA (molecular mechanics-generalized born surface area) method. The results showed that Gni_Epd exhibited the lowest binding free energy score of -64.03 kcal/mol for NAD^+, indicating strong binding, while the binding energy of E4P (-13.11 kcal/mol) was slightly higher than that of Eco_Epd (-12.54 kcal/mol), suggesting some stabilization of E4P.

Among the other sources, Ame_Epd led to a decrease in yield as the binding energy values for both NAD^+ and E4P increased. This suggests that the binding of both NAD^+ and E4P may contribute to the enzymatic activity. However, for Csa_Epd, Pfl_Epd, and Hel_Epd, the binding affinity of NAD^+ appeared to be more advantageous. Although Csa_Epd showed improved binding ability for E4P (-24.04 kcal/mol), the binding ability of NAD^+ (-48.58 kcal/mol) did not improve, resulting in no increase in yield. On the other hand, the other two sources had high binding energy values for E4P (Pfl_Epd: -12.10 kcal/mol, Hel_Epd: -4.40 kcal/mol) but low binding energy values for NAD^+ (Pfl_Epd: -58.81 kcal/mol, Hel_Epd: -62.28 kcal/mol), which did contribute to the increased yield.

Considering the relative sufficiency of E4P supply, it has been observed that the yield of L-phenylalanine synthesized from E4P can reach over 90 g/L without altering the precursor synthesis pathway [12]. Hence, we speculate that the current yield level already has an adequate supply of E4P. Thus, even if some E4P may not always be stably bound in the catalytic region, the sufficient E4P supply can compensate for this deficiency, making the supply of NAD^+ more crucial. However, effective binding of E4P may become more significant in later stages when the yield is further increased and a larger amount of E4P substrate needs to be consumed.

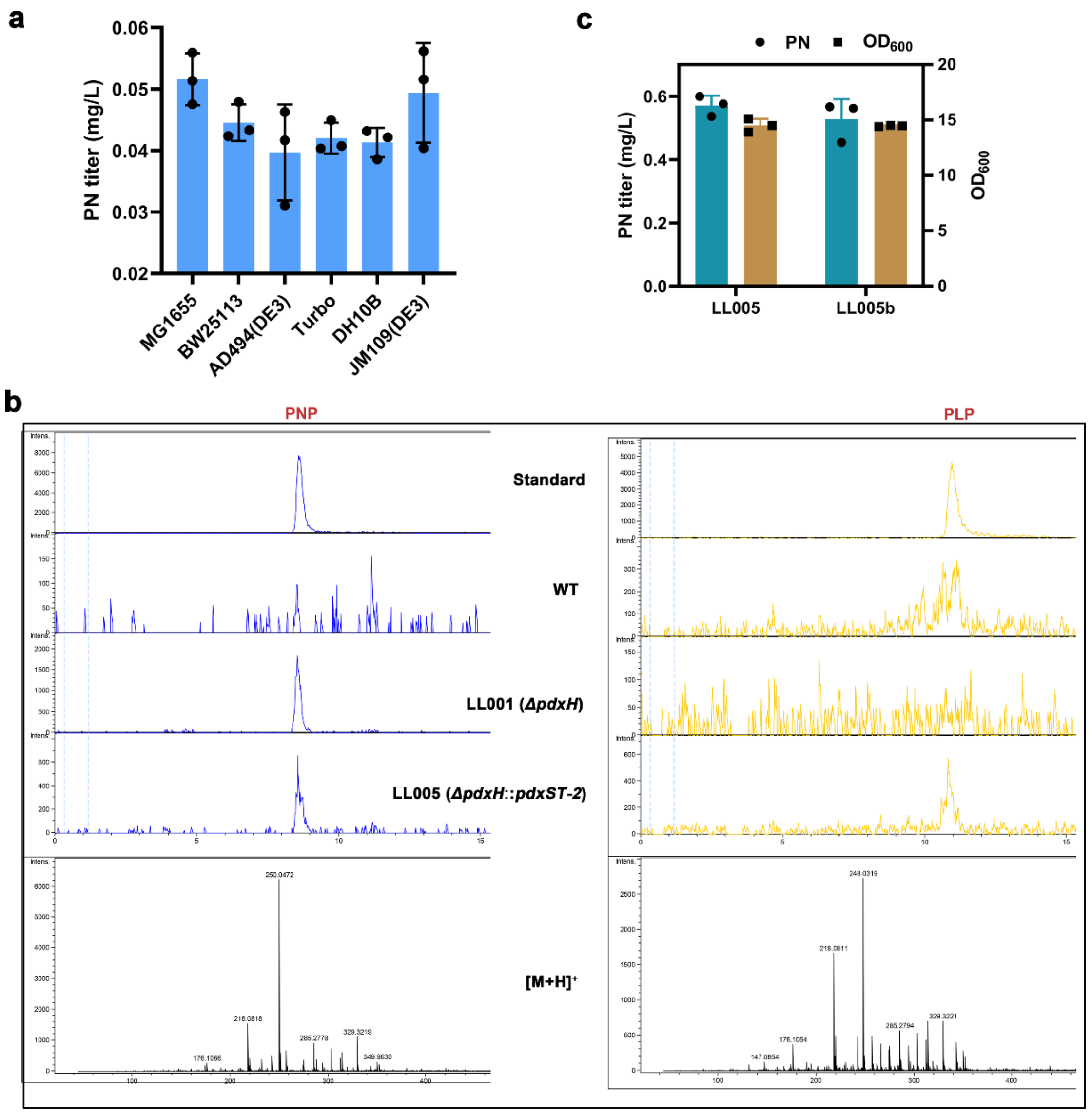

Supplementary Fig. 1. The screening of wild-type (WT) *E. coli* and the level of PNP or PLP in WT and the mutants engineered by parallel pathway. a The PN titer of different WT *E. coli* strains cultured in 24 deep well plates. **b** The LC-MS spectra of PNP and PLP produced by fermentation of *E. coli* mutants engineered by parallel pathway. The $[M+H]^+$ ions of PNP at m/z 250.0472 (left panel) and PLP at m/z 248.0319 (right panel). MicrOTOF and DataAnalysis was used to collect and analyze the HPLC-MS data, respectively. **c** The PN titer and cell growth of LL005 and LL005b (LL005 *Δpta*). **a, c** Data are presented as mean values ± SD from three independent biological replicates (n = 3), the circles or squares represent individual data points. Source data are provided as a Source Data file.

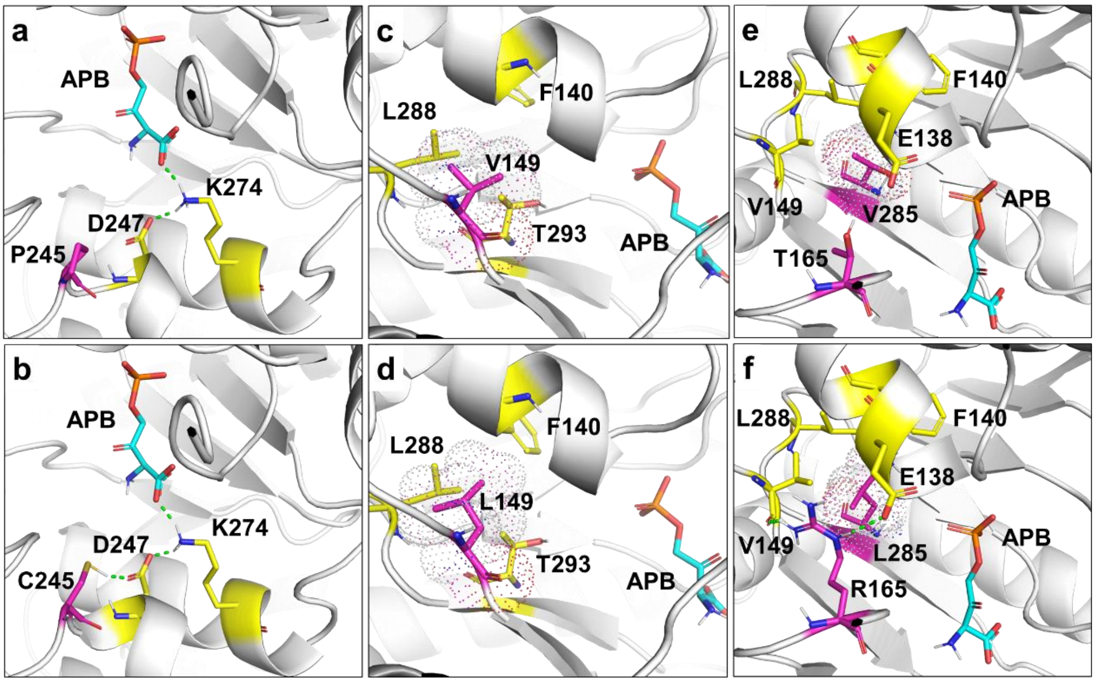

Supplementary Fig. 2. Analysis of the interaction networks of PdxA mutants and WT. a/b Changes of hydrogen bonding network before and after P245C mutation; **c/d** Changes of hydrophobic interactions before and after V149L mutation; **e/f** Changes of hydrogen bonding and hydrophobic interactions before and after T165R/V285L mutation. Mutated residues are shown as magenta sticks, substrates are cyan, and unmutated residues are yellow. Green dashed lines indicate hydrogen-bonding interactions. Dots represent van der Waals accessible regions of the residues. APB, 2-amino-3-oxo-4-phosphonooxybutyrate.

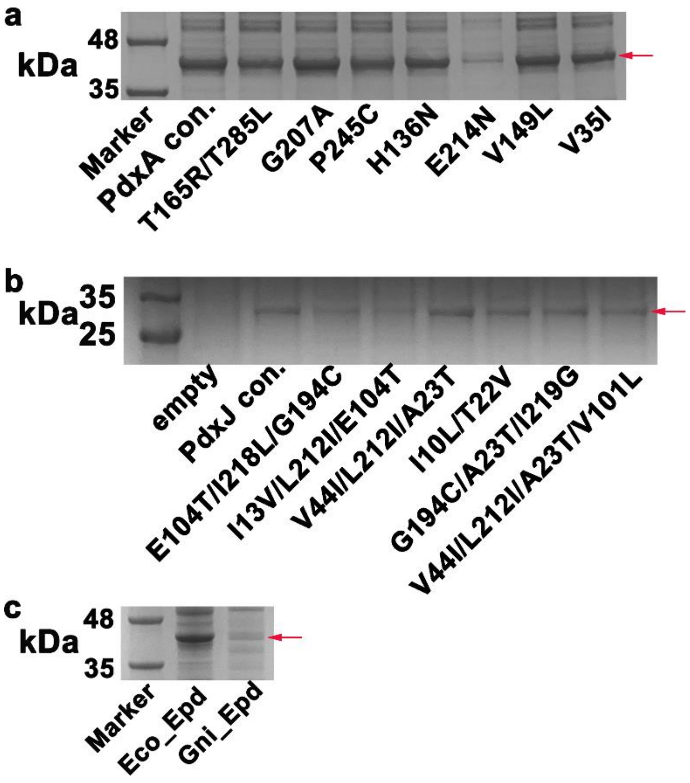

Supplementary Fig. 3. The expression of PdxA, PdxJ, Epd, and their mutant were identified through SDS-PAGE analysis. a The expression of PdxA and its mutants. **b** The expression of PdxJ and its mutants. **c** The expression of Eco_Epd and Gni_Epd. The target protein is marked with a red arrow. PdxA con. and PdxJ con. represent the native proteins. Source data are provided as a Source Data file.

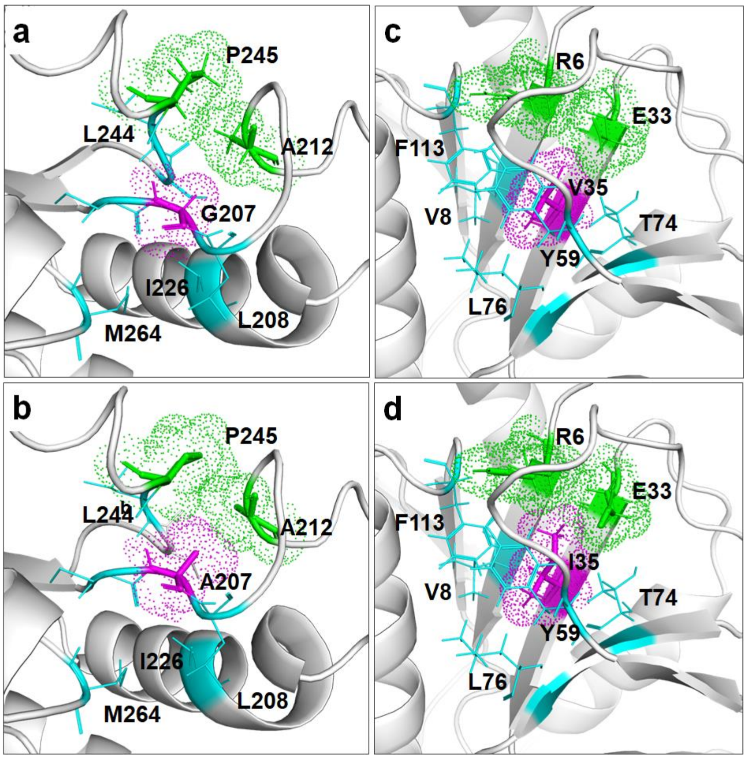

Supplementary Fig. 4. Structural analysis of G207A and V35I compared to the WT. a/b Changes in the van der Waals range of G207A before and after mutation. A207 fills the cavity structure that was not saturated by G207, P245 and A212. **c/d** Changes in the van der Waals range of V35I before and after mutation. I35 fills the cavity structure that was not saturated by V35, R6 and E33. Dots indicate the van der Waals accessible surface area positions of the residues. The residues are presented as sticks.

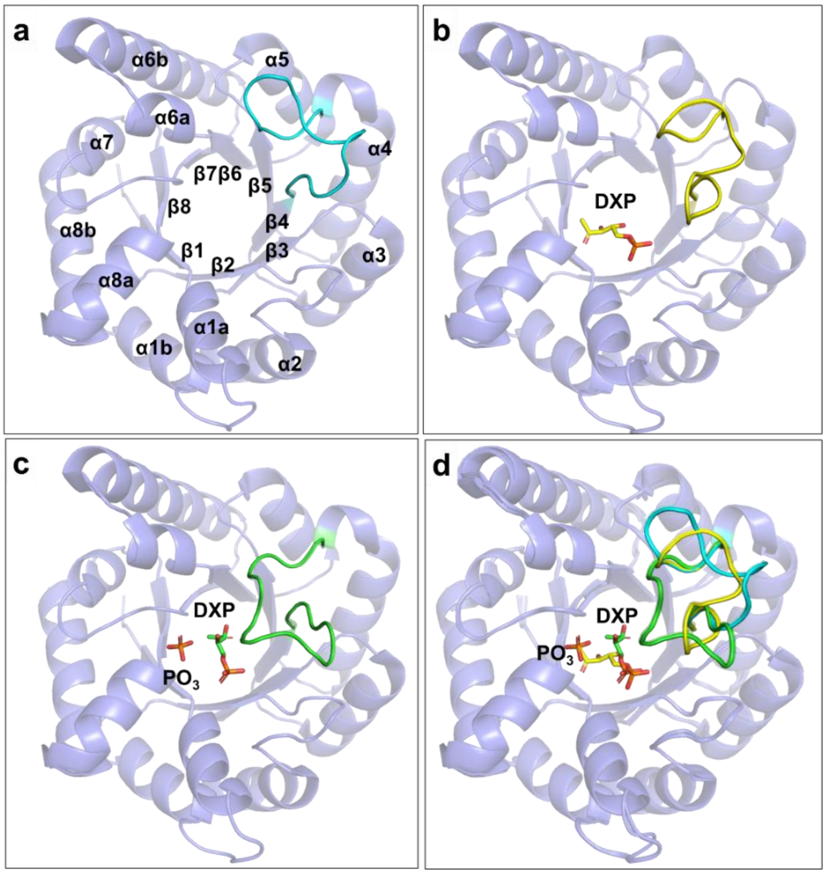

Supplementary Fig. 5. Three apparent structurally and functionally distinct states of PdxJ. a Resting, open state. **b** Partially open state with bound DXP alone. **c** Closed state with bound DXP and inorganic phosphate, a surrogate of AHP positioned to react. **d** Alignment of these three states. The different loop structures were colored cyan, yellow, and green in the open state, partially open state, and closed state, respectively.

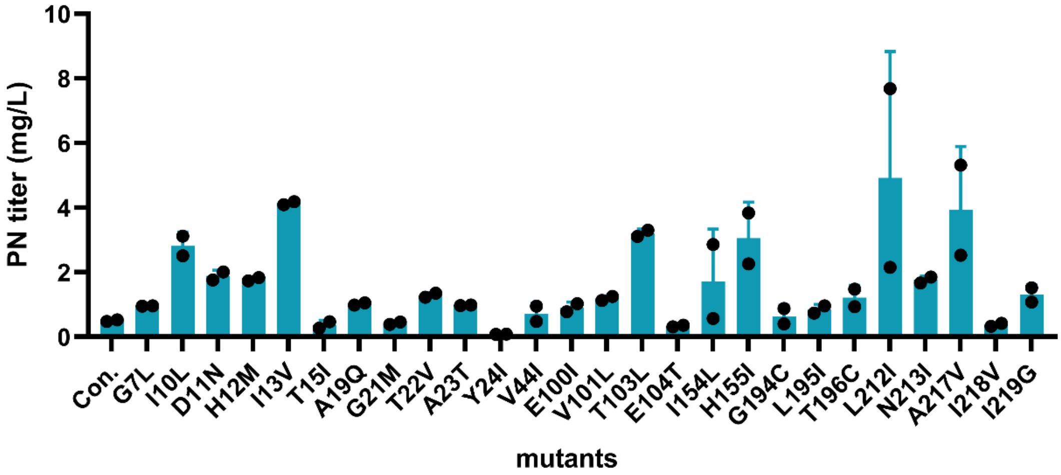

Supplementary Fig. 6. The PN titer of the *pdxJ* overexpression mutants with single amino acid substitution. The strain with native *pdxJ* overexpression was used as the control (Con.). Data are presented as mean values ± SD from two independent biological replicates (n = 2). Source data are provided as a Source Data file.

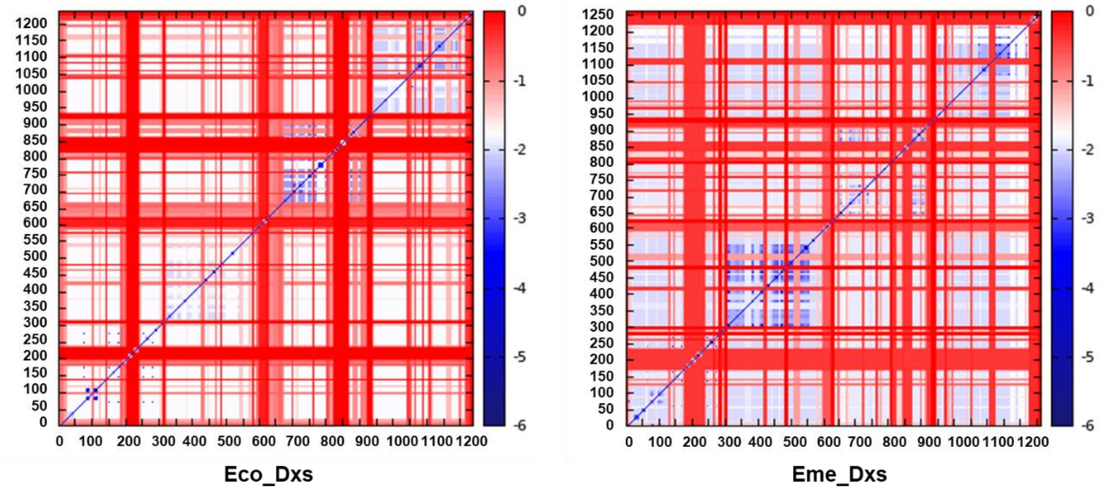

Supplementary Fig. 7. The stability map (dimer protein used) of Eco_Dxs and Eme_Dxs. Red colors indicate pairs of residues where no or only a weak rigid contact exists. Blue colors indicate strong rigid contacts.

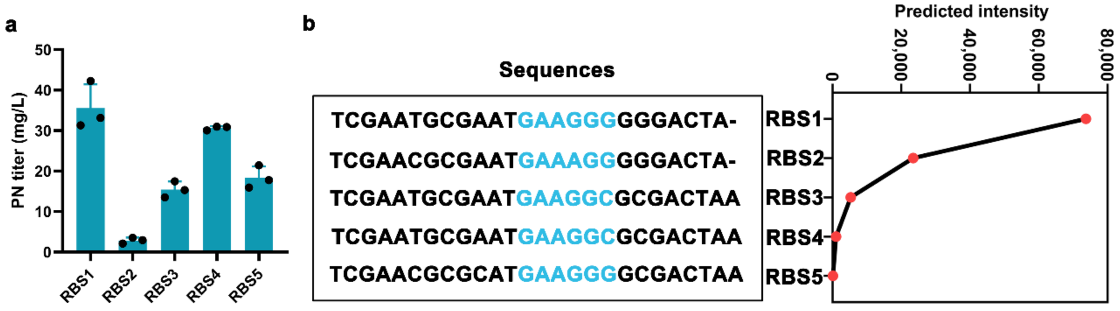

Supplementary Fig. 8. The RBS optimization of PdxB in the multi-module optimization strategy. a The PN titer of mutants with ribosomal binding site (RBS) optimization of PdxB. Data are presented as mean values ± SD from three independent biological replicates (n = 3), the circles or squares represent individual data points. **b** The sequences and the intensity of the RBS. Source data are provided as a Source Data file.

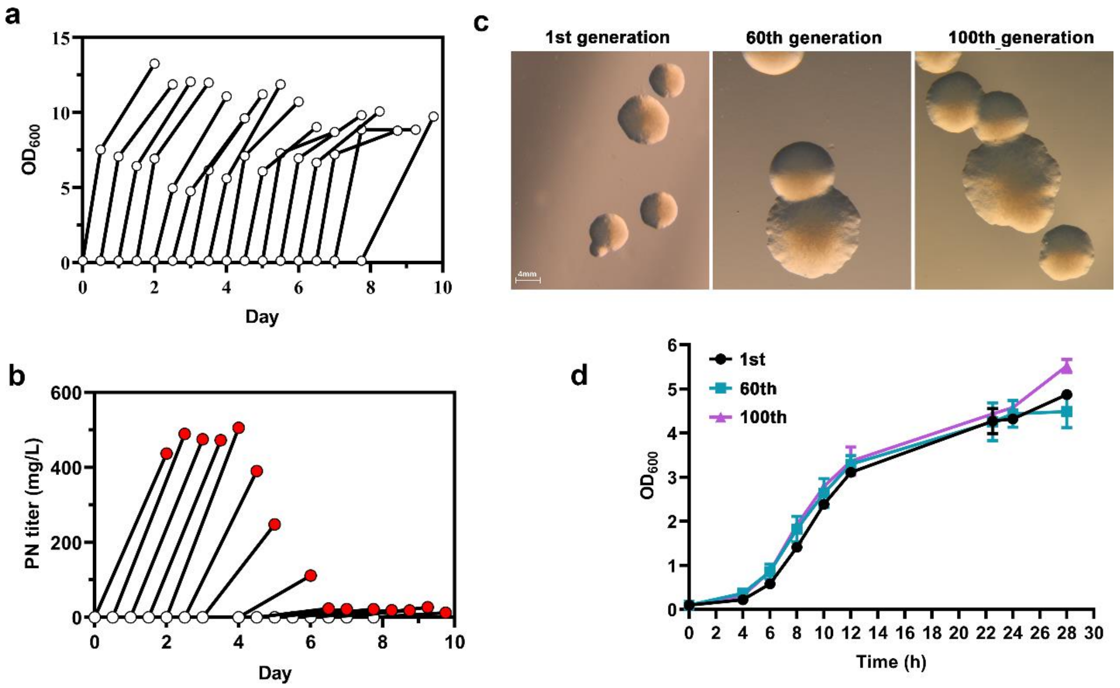

Supplementary Fig. 9. Genetic stability of the engineered best-producing strain. a, **b** The cell growth (OD_{600}) and PN titer of the evolved strains of LL388, which were sequentially transferred in FM1.4 medium. **c** The colony heterogeneities of the parental strain and the evolved mutants. **d** The growth curves of the cells of the 1st, 60th and 100th generations. The 60th and 100th generations were about the fifth- and eighth-day passaging, respectively. Source data are provided as a Source Data file.

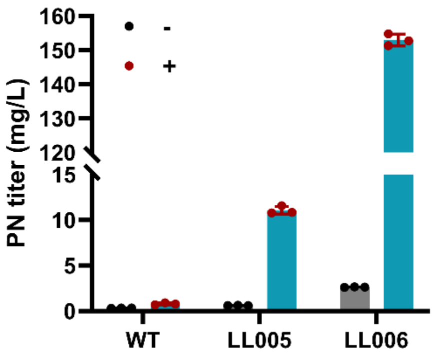

Supplementary Fig. 10. The PN titer of the mutants which introduced R42 and P3A2J1 plasmids into WT, LL005, and LL006. The black dots indicate the original strain, and the red dots indicate that the plasmids were introduced into the strains. Data are presented as mean values ± SD from three independent biological replicates (n = 3). Source data are provided as a Source Data file.

Supplementary Table 1. RBS sequences of *pdxST* operon.

**RBS name**	**Sequences (5’-3’)**	**Translation initiation rate (au)**
R1a-*pdxS*	CAATCCTAGAAAAGGAGGTAGTCTA	19097.97
R1b-*pdxT*	TTTGTAACTAGGAGGAGGGATTATA	13663.78
R2a-*pdxS*	AAAAACAAAAGACGGAGGTCAAATT	14629.87
R2b-*pdxT*	TTGGTTTACCCATACGGAGGAGGGC	9170.75

Supplementary Table 2. The separately calculated binding energy for the substrate (E4P) and coenzyme (NAD^+) using the MMGBSA method in the pre-catalytic state.

Epd name	Binding energy with E4P (kcal/mol)	Binding energy with NAD^+ (kcal/mol)
Eco_Epd	-12.54	-53.34
Plu_Epd	-13.25	54.76
Xne_Epd	-11.51	-51.09
Psn_Epd	-15.28	-51.12
Ype_Epd	-17.27	-54.41
Eam_Epd	-15.74	-49.08
Csa_Epd	-24.04	-48.58
Ame_Epd	-9.72	-49.79
Gni_Epd	-13.11	-64.04
Pfl_Epd	-12.10	-58.81
Hel_Epd	-4.40	-62.28
Mpo_Epd	-0.64	-53.87

Supplementary Table 3. The criteria for screening heterologous Dxs enzymes.

Dxs source	Criteria of Dxs screening
E. coli	G3P: k_{cat} = 1.38 s^{-1}, *K*m = 0.0142-0.042 mM, k_{cat}/*K*m = 13.67-95 [s^{-1}/mM]; Pyr: k_{cat} = 2.6 s^{-1}, *K*m = 0.0011-0.049 mM; k_{cat}/*K*m = 32.2 [s^{-1}/mM] [13-15].
Deinococcus radiodurans	G3P: k_{cat} = 2.57-7.9 s^{-1}, *K*m = 0.0235-0.05 mM, k_{cat}/*K*m = 113.3 [s^{-1}/mM]; Pyr: k_{cat} = 2.57-7.4 s^{-1}, *K*m = 0.049-0.28 mM, k_{cat}/*K*m = 26-53.3 [s^{-1}/mM] [16,17].
Agrobacterium tumefaciens	G3P: k_{cat} = 26.8 s^{-1}, *K*m = 0.0232 mM; Pyr: k_{cat} = 26.8 s^{-1}, *K*m = 0.04 mM [18].
Rhodobacter capsulatus	G3P: k_{cat} = 1.9 s^{-1}, *K*m = 0.068 mM; Pyr: k_{cat} = 1.9 s^{-1}, *K*m = 0.44 mM [19].
Rhodobacter sphaeroides	Overexpression of Rsp_Dxs to enhance production of CoQ_{10}, lycopene, β-carotene etc. [20-22].
B. subtilis	the Gram-positive bacteria; Bsu_Dxs overexpression to enhance production of menaquinone-7, menaquinone-4, isoprene etc. [23-26].
E. meliloti	a natural overproducer of vitamin B_6 [27].

Supplementary Table 4. Promoter sequences of *pdxA-pdxJ* operon.

**Promoter name**	**Sequence**	**Transcription initiation rate (au)**
P_{J23119}	TTGACAGCTAGCTCAGTCCTAGGTATAATGCTAGC	6243.28
P2	TTGACAGCTAGCTCAGTCCTAGGTAATGCTAGC	2831.99
P3	AGGACAGCTAGCTCAGTCCTAGGTTAATGCTAG	1329.07
P4	CATGTAGCGTCTATTTTTCTGTCCCTAGATTGTGTG AGAACCGGAGTTCACTGTCCGGGC	1007.01

Supplementary Table 5. The cellular amounts of the overproduced proteins (Mol proportion).

Protein name	LL006b-10.5 OD_{600} (Shake flask)	LL388-10 OD_{600} (Shake flask)	LL388-10 OD_{600} (5L bioreactor)	LL388-26 OD_{600} (5L bioreactor)
Gni_Epd	ND[1]	0.925%	0.564%	0.688%
PdxB	0.018%	1.021%	0.838%	1.202%
SerC	0.036%	0.322%	0.474%	0.347%
Eme_Dxs	ND[1]	0.156%	0.067%	0.089%
PdxA2	0.002%	0.945%	1.996%	1.887%
PdxJ1	0.014%	3.706%	6.279%	4.275%
Eme_PdxP	0.145%	0.071%	0.026%	0.123%
Bsu_PdxS	0.240%	0.491%	0.484%	0.880%
Bsu_PdxT	0.052%	0.123%	0.240%	0.147%

[1]ND, Not detected.

Supplementary Table 6. Plasmids used in this study.

Plasmids	Description	Source
pRSFDuet-1	RSF origin, T7 promoter, two multiple cloning sites, $Kana^r$	Our lab
p15ASI	tac promoter, p15A ori, Cml^r	Our lab
pCas9	araBAD promoter, repA101 ori, Amp^r	Our lab
pRSFDuet-1_*pdxA*	pRSFDuet-1 containing *pdxA* or *pdxA* mutations	This study
pRSFDuet-1_*pdxJ*	pRSFDuet-1 containing *pdxJ* or *pdxJ* mutations	This study
R42	p15ASI containing Ptac-*epd* (Gni)-*pdxB* (Eco) -*dxs* (Eme)-$P_{J231119}$ -*serC* (Eco)	This study
P3A2J1	pRSFDuet-1-P3-*pdxA2*-*pdxJ1*	This study

Supplementary references

1. Liu, Y. *et al.* Genetic engineering of *Escherichia coli* to improve L-phenylalanine production. *BMC Biotechnol.* **18,** 5 (2018).
2. Chen, Y. *et al.* Proteomic analysis identifies prolonged disturbances in pathways related to cholesterol metabolism and myocardium function in the COVID-19 recovery stage. *J. Proteome Res.* **20,** 3463-3474 (2021).
3. Schwanhausser, B. *et al.* Global quantification of mammalian gene expression control. *Nature* **473,** 337-342 (2011).
4. Krey, J. F. *et al.* Accurate label-free protein quantitation with high- and low-resolution mass spectrometers. *J. Proteome Res.* **13,** 1034-1044 (2014).
5. Araz *et al.* Fast, efficient generation of high-quality atomic charges. AM1-BCC model: I. Method. *J. Comput. Chem.* **21,** 132-146 (2000).
6. Jorgensen, W. L., Chandrasekhar, J., Madura, J. D., Impey, R. W. & Klein, M. L. Comparison of simple potential functions for simulating liquid water. *J.chem.phys* **79,** 926-935 (1998).
7. Pearlman, D. A. *et al.* AMBER, a package of computer programs for applying molecular mechanics, normal mode analysis, molecular dynamics and free energy calculations to simulate the structural and energetic properties of molecules. *Comput. Phys. Commun.* **91,** 1-41 (1995).
8. Andersen, H. C. Rattle: A "velocity" version of the shake algorithm for molecular dynamics calculations. *J. Comput. Phys.* **52,** 24-34 (1983).
9. Chow, K. H. & Ferguson, D. M. Isothermal-isobaric molecular dynamics simulations with Monte Carlo volume sampling. *Comput. Phys. Commun.* **91,** 283-289 (1995).
10. Le Grand, S., Gotz, A. W. & Walker, R. C. SPFP: Speed without compromise-A mixed precision model for GPU accelerated molecular dynamics simulations. *Comput. Phys. Commun.* **184,** 374-380 (2013).
11. Roe, D. R. & Cheatham, T. E. PTRAJ and CPPTRAJ: software for processing and analysis of molecular dynamics trajectory data. *J. Chem. Theory Comput.*

9, 3084-3095 (2013).

12. Ding, D. *et al.* Biosensor-based monitoring of the central metabolic pathway metabolites. *Biosens. Bioelectron.* **167,** 112456 (2020).
13. DeColli, A. A., Zhang, X., Heflin, K. L., Jordan, F. & Freel Meyers, C. L. Active site histidines link conformational dynamics with catalysis on anti-infective target 1-deoxy-d-xylulose 5-phosphate synthase. *Biochemistry* **58,** 4970-4982 (2019).
14. Brammer, L. A. & Meyers, C. F. Revealing substrate promiscuity of 1-deoxy-D-xylulose 5-phosphate synthase. *Org. Lett.* **11,** 4748-4751 (2009).
15. Brammer, L. A., Smith, J. M., Wade, H. & Meyers, C. F. 1-Deoxy-D-xylulose 5-phosphate synthase catalyzes a novel random sequential mechanism. *J. Biol. Chem.* **286,** 36522-36531 (2011).
16. White, J. K., Handa, S., Vankayala, S. L., Merkler, D. J. & Woodcock, H. L. Thiamin diphosphate activation in 1-deoxy-D-xylulose 5-phosphate synthase: insights into the mechanism and underlying intermolecular interactions. *J. Phys. Chem. B* **120,** 9922-9934 (2016).
17. Basta, L. A. B., Patel, H., Kakalis, L., Jordan, F. & Meyers, C. L. F. Defining critical residues for substrate binding to 1-deoxy-D-xylulose 5-phosphate synthase--active site substitutions stabilize the predecarboxylation intermediate C2α-lactylthiamin diphosphate. *Febs J.* **281,** 2820-2837 (2014).
18. Lee, J. K., Oh, D. K. & Kim, S. Y. Cloning and characterization of the *dxs* gene, encoding 1-deoxy-d-xylulose 5-phosphate synthase from *Agrobacterium tumefaciens*, and its overexpression in *Agrobacterium tumefaciens*. *J. Biotechnol.* **128,** 555-566 (2007).
19. Eubanks, L. M. & Poulter, C. D. *Rhodobacter capsulatus* 1-deoxy-D-xylulose 5-phosphate synthase: steady-state kinetics and substrate binding. *Biochemistry* **42,** 1140-1149 (2003).
20. Qiang, S. *et al.* Elevated beta-carotene synthesis by the engineered *Rhodobacter sphaeroides* with enhanced CrtY expression. *J. Agric. Food Chem.* **67,** 9560-9568 (2019).

21. Zhu, Y. *et al.* Enhanced CoQ_{10} production by genome modification of *Rhodobacter sphaeroides* via Tn7 transposition. *FEMS Microbiol. Lett.* **369** (2022).

22. Su, A. *et al.* Metabolic redesign of *Rhodobacter sphaeroides* for lycopene production. *J. Agric. Food Chem.* **66,** 5879-5885 (2018).

23. Zhao, Y. *et al.* Biosynthesis of isoprene in *Escherichia coli* via methylerythritol phosphate (MEP) pathway. *Appl. Microbiol Biotechnol.* **90,** 1915-1922 (2011).

24. Ding, X. *et al.* Bottom-up synthetic biology approach for improving the efficiency of menaquinone-7 synthesis in *Bacillus subtilis*. *Microb. Cell. Fact.* **21,** 101 (2022).

25. Yuan, P. *et al.* Combinatorial engineering for improved menaquinone-4 biosynthesis in *Bacillus subtilis*. *Enzyme Microb. Technol.* **141,** 109652 (2020).

26. Abdallah, II, Pramastya, H., van Merkerk, R., Sukrasno & Quax, W. J. Metabolic engineering of *Bacillus subtilis* toward taxadiene biosynthesis as the first committed step for taxol production. *Front Microbiol.* **10,** 218 (2019).

27. Tazoe, M., Ichikawa, K. & Hoshino, T. Production of Vitamin B6 in Rhizobium. *Biosci Biotechnol Biochem* **63,** 1378-1382 (1999).
