# Supplementary material for: Protein engineering and iterative multimodule optimization for vitamin B6 production in Escherichia coli
Source: Nat Commun. 2023 Aug 31;14:5304. doi: 10.1038/s41467-023-40928-0 (PMC10471632; doi:10.1038/s41467-023-40928-0)
Supplement: Supplementary file 3 — Description of Additional Supplementary Files [file 41467_2023_40928_MOESM3_ESM.pdf]

Description of Additional Supplementary Files

Supplementary Data 1: PdxA mutants from activity design;

Supplementary Data 2: PdxA mutants from stability design;

Supplementary Data 3: PdxJ mutants from activity design;

Supplementary Data 4: Strains used in this study;

Supplementary Data 5: Primers used in this study;

Supplementary Data 6: Sequences of the synthetic genes used in this study.
