# Supplementary material for: Protein engineering and iterative multimodule optimization for vitamin B6 production in Escherichia coli
Source: Nat Commun. 2023 Aug 31;14:5304. doi: 10.1038/s41467-023-40928-0 (PMC10471632; doi:10.1038/s41467-023-40928-0)
Supplement: Supplementary file 10 — Reporting Summary [file 41467_2023_40928_MOESM10_ESM.pdf]

nature portfolio

Corresponding author(s): Dawei Zhang

Last updated by author(s): Aug 8, 2023

Reporting Summary

Nature Portfolio wishes to improve the reproducibility of the work that we publish. This form provides structure for consistency and transparency in reporting. For further information on Nature Portfolio policies, see our Editorial Policies and the Editorial Policy Checklist.

Statistics

For all statistical analyses, confirm that the following items are present in the figure legend, table legend, main text, or Methods section.

n/a	Confirmed	
☐	☒	The exact sample size (*n*) for each experimental group/condition, given as a discrete number and unit of measurement
☐	☒	A statement on whether measurements were taken from distinct samples or whether the same sample was measured repeatedly
☐	☒	The statistical test(s) used AND whether they are one- or two-sided<br>*Only common tests should be described solely by name; describe more complex techniques in the Methods section.*
☒	☐	A description of all covariates tested
☒	☐	A description of any assumptions or corrections, such as tests of normality and adjustment for multiple comparisons
☐	☒	A full description of the statistical parameters including central tendency (e.g. means) or other basic estimates (e.g. regression coefficient) AND variation (e.g. standard deviation) or associated estimates of uncertainty (e.g. confidence intervals)
☐	☒	For null hypothesis testing, the test statistic (e.g. *F*, *t*, *r*) with confidence intervals, effect sizes, degrees of freedom and *P* value noted<br>*Give P values as exact values whenever suitable.*
☒	☐	For Bayesian analysis, information on the choice of priors and Markov chain Monte Carlo settings
☒	☐	For hierarchical and complex designs, identification of the appropriate level for tests and full reporting of outcomes
☒	☐	Estimates of effect sizes (e.g. Cohen's *d*, Pearson's *r*), indicating how they were calculated

Our web collection on statistics for biologists contains articles on many of the points above.

Software and code

Policy information about availability of computer code

Data collection	Chromeleon (version 7) was used to collect the HPLC data. PyMOL (Version 2.3.3) and MEGA (Version 11.0.11) were used to plot images. MicrOTOF was used to collect the HPLC-MS data. Gen5 (version 3.05) was used to collect the enzyme kinetics data.
Data analysis	Chromeleon (version 7) was used to analyze the HPLC data. DataAnalysis was used to analyze the HPLC-MS data. OriginPro (version 9.1) and GraphPad Prism (version 8) were used to analyze the enzyme kinetics data and statistical data. Amber18 was used to calculate Molecular dynamics data. Rosetta3.13 was used to simulate mutations and generate mutant structures. Codes were written with Python3.7.

For manuscripts utilizing custom algorithms or software that are central to the research but not yet described in published literature, software must be made available to editors and reviewers. We strongly encourage code deposition in a community repository (e.g. GitHub). See the Nature Portfolio guidelines for submitting code & software for further information.

Data

Policy information about availability of data

All manuscripts must include a data availability statement. This statement should provide the following information, where applicable:

- Accession codes, unique identifiers, or web links for publicly available datasets
- A description of any restrictions on data availability
- For clinical datasets or third party data, please ensure that the statement adheres to our policy

Source data are provided with this paper. All data presented in graphs within the Figures and Table 1 are provided as a Source Data file. The processed proteomic

data are available at Supplementary Table 5 and the raw proteomic data are available from the corresponding author on request. Sequence data in this article can be found in the Supplementary Data 6 file.

Human research participants

Policy information about studies involving human research participants and Sex and Gender in Research.

Reporting on sex and gender	Our article contains no sex/gender related topics, therefore such information is irrelevant to our article and has not been collected.
Population characteristics	Our article contains no sex/gender related topics, therefore such information is irrelevant to our article and has not been collected.
Recruitment	No recruitment of participants occurred during the experiment in our article.
Ethics oversight	Not applicable

Note that full information on the approval of the study protocol must also be provided in the manuscript.

Field-specific reporting

Please select the one below that is the best fit for your research. If you are not sure, read the appropriate sections before making your selection.

☒ Life sciences ☐ Behavioural & social sciences ☐ Ecological, evolutionary & environmental sciences

For a reference copy of the document with all sections, see nature.com/documents/nr-reporting-summary-flat.pdf

Life sciences study design

All studies must disclose on these points even when the disclosure is negative.

Sample size	No statistical method was used to determine sample size. As indicated in the text, all experiments were performed from two or three single reaction groups.
Data exclusions	No data were excluded from the analyses.
Replication	Two or three independent replicates were performed for all experiments. All attempts at replication were successful.
Randomization	Randomization is not relevant in this study, as enzymes and engineered strains are unique and have definite functions.
Blinding	Blinding is not relevant in this study.

Reporting for specific materials, systems and methods

We require information from authors about some types of materials, experimental systems and methods used in many studies. Here, indicate whether each material, system or method listed is relevant to your study. If you are not sure if a list item applies to your research, read the appropriate section before selecting a response.

Materials & experimental systems

n/a	Involved in the study	
☒	☐	Antibodies
☒	☐	Eukaryotic cell lines
☒	☐	Palaeontology and archaeology
☒	☐	Animals and other organisms
☒	☐	Clinical data
☒	☐	Dual use research of concern

Methods

n/a	Involved in the study	
☒	☐	ChIP-seq
☒	☐	Flow cytometry
☒	☐	MRI-based neuroimaging
